# Antioxidant, Physicochemical, and Cellular Secretion of Glucagon-Like Peptide-1 Properties of Oat Bran Protein Hydrolysates

**DOI:** 10.3390/antiox9060557

**Published:** 2020-06-26

**Authors:** Mallory E. Walters, William G. Willmore, Apollinaire Tsopmo

**Affiliations:** 1 Food Science and Nutrition Program, Department of Chemistry, Carleton University, 1125 Colonel By Drive, Ottawa, ON K1S 5B6, Canada; MalloryWalters@cmail.carleton.ca; 2 Department of Biology, Carleton University, 1125 Colonel By Drive, Ottawa, ON K1S 5B6, Canada; Bill.Willmore@carleton.ca; 3 Institute of Biochemistry, Carleton University, 1125 Colonel By Drive, Ottawa, ON K1S 5B6, Canada

**Keywords:** oat proteins, antioxidant, anti-diabetic, colorectal cells

## Abstract

The aim of this work was to determine the physicochemical and biological activities of hydrolyzed proteins from sonicated oat brans. In addition to the control bran sample, two types of pre-treatment procedures—namely, ultrasonic bath and probe-type sonication—were performed to extract proteins, followed by hydrolysis with various proteases. Physicochemical analyses showed that Flavourzyme-hydrolysates had greater amounts of aromatic amino acids, Papain-hydrolysates low surface charges (−0.78 to −1.32 mV) compared to the others (−3.67 to −9.17 mV), and Alcalase-hydrolysates a higher surface hydrophobicity. The hydrolysates had good radical scavenging activities but, as the ultrasonic pre-treatment of the brans showed, in certain cases there was a reduction in activities of up to 22% for ROO^•^ and HO^•^ and 15% for O_2_^•−^ radicals. In anti-diabetic tests, the maximum inhibition of α-amylase was 31.8%, while that of dipeptidyl peptidase-4 was 53.6%. In addition, the secretion of glucagon-like peptide-1 in NCI-H716 cells was enhanced by 11.5% in the presence of hydrolysates.

## 1. Introduction

Oats (*Avena sativa)* are a rich source of nutrients, including lipids, proteins, and vitamins as well as dietary fibers and polyphenols, which contributes to their increased use worldwide [1]. The increased consumption of oat products is also due consumer awareness of the benefits that oats and other cereals can provide in the prevention and management of chronic conditions such as diabetes, hypertension, and cardiovascular diseases [2]. According to the literature, the mechanism of protection can be through the reduction in oxidative stress; the lowering of cholesterol levels; or the regulation of hormones, genes, and enzymes [3]. One of these conditions, type 2 diabetes mellitus (T2DM), is estimated to affect 8.8% of the global population by 2035 [4]. In people with T2DM, a lack of insulin sensitivity leads to high plasma glucose levels. Current medications work by increasing the release of insulin from the beta cells in the pancreas (e.g., sulfonylureas), or by decreasing the amount of fatty acids present in the circulation, thereby making cells more dependent on glucose for energy (e.g., thiazolindinediones). Medications for T2DM also work by decreasing hepatic glucose production and increasing insulin-mediated glucose uptake (e.g., metformin), as well as by inhibiting enzymes that catalyze the degradation of lysosomal glycogen (e.g., alglucosidase alfa) or the degradation of insulinotropic hormones [5]. Although these drugs are effective for mitigating the symptoms of T2DM, there is evidence that some might lead to hypoglycemia or increase the risk of weight gain and heart failure [6]. There is thus a need for natural alternatives, some of which include hydrolyzed proteins and peptides from foods. In this regard, recent research has demonstrated the potential of food protein hydrolysates to regulate blood glucose levels through the inhibition of polysaccharide degrading enzymes such as amylase and glucosidase [3]. Another target has been the inhibition of dipeptidyl peptidase-4 (DDP-4), a ubiquitously expressed enzyme that cleaves the incretin hormones glucagon-like peptide 1 (GLP-1) and glucose-dependent insulinotropic protein (GIP), which are responsible for stimulating up to 70% of the post-prandial insulin response [4]. Hydrolyzed proteins and peptides with DPP-4 inhibitory activities have been reported from rice, wheat, amaranth, and soybean [4,7]. Other works have reported the α-amylase and α-glucosidase inhibitory activities of hydrolyzed cereal proteins [3,8]. Protein hydrolysates possess other properties, such as antioxidant, cytoprotective, and anti-hypertensive [3], all of which can be affected by the extraction procedure.

The use of ultrasounds can disrupt the food matrix and facilitate the extraction of proteins, as reported, for example, in walnut [9]. Ultrasound treatments can also affect the secondary structures of proteins, which can affect their behavior in the presence of proteases and, consequently, the biological activity of the hydrolysates. Data on the application of ultrasounds to extract proteins in oats are not available. The aim of this work was therefore: (1) to test the effect of the sonication of protein proteins extraction yields from oat brans; (2) digest the proteins with various proteases and evaluate their antioxidant activities and anti-diabetic properties (inhibition of α-amylase and DPP-4, cellular secretion of GLP-1).

## 2. Methodology

### 2.1. Materials and Chemicals

Medium bran oat flour (i.d. 112-001) with a particle size percentage distribution of 2.00 mm (0.8%), 0.841 mm (61.5%), 0.595 mm (32.1%), 0.420 mm (5.0%), and Pan (0.6%) was donated by Richardson Milling (Portage La Prairie, MB, Canada). The enzymes (α-amylase, Flavourzyme^®^, Alcalase^®^, and Papain, sodium tartrate, sodium dodecyl sulfate, cupric sulfate pentahydrate, Trolox, 1,10-phenanthroline, iron(II) sulfate heptahydrate, hydrogen peroxide (H_2_O_2_), Tris-HCl, Tris-Base, potassium bromide, pyrogallol, reduced glutathione, L-serine, 3,5-dinitrosalicylic acid, sodium potassium phosphate tartrate, starch, and bovine serum albumin (BSA) were purchased from Sigma-Aldrich Ltd. (Oakville, Ontario, Canada). The solvents, including concentrated hydrochloric acid, methanol and Folin-Ciocalteau Phenol reagent, fluorescein, and 2,2’-azobis(2-amidinopropane) dihydrochloride (AAPH), were purchased from Fisher Scientific Co. (Nepean, Ontario, Canada).

### 2.2. Protein Extraction

The medium oat bran flour was defatted using n-hexane, stirred for one hour with a 1:4 ratio (*w/v*), and dried for 24 h in a fume hood. The defatted flour was then mixed with 0.02 M of NaOH (1:10 ratio (*w/v*)) and adjusted to a pH of 9.5. For the ultrasonic bath treatment, the slurry was placed in an ultrasonic cleaner (Model FS30, 100 W, 42 kHz, Fisher Scientific Co., Nepean, Ontario, Canada) bath for 5 min with continuous manual stirring. The ultrasonic probe sonication was performed with a UIP500hdT model processor (Heilscher Ultrasound Technologies, Germany). The probe was immersed in the slurry at 1.5 cm and sonicated at 20 kHz, 100 W for 5 min. The control slurry did not undergo ultrasonic treatment. All three slurries were then shaken in an incubator (140 rpm) for 2 h at 25 °C, after which they were centrifuged (2500× *g*, 20 min, 4 °C). The supernatants were collected, adjusted to pH 4.5, and centrifuged once more (10,000× *g*, 40 min, 4 °C) to precipitate and recover the proteins, which were then freeze dried and stored at −20 °C.

### 2.3. Gel Electrophoresis and Mass Sspectrometry Analysis of Proteins

The distribution of the molecular weights of the proteins in the samples was determined using sodium dodecyl sulfate polyacrylamide gel electrophoresis (SDS-PAGE), using previously published methodologies [10]. One set of samples were dissolved in a reducing buffer (0.125M Tris-HCl pH 6.8, 4% *w/v* SDS, 20% *v/v* glycerol, and 0.5% 2-mercaptoethanol), while the other set of samples was dissolved in buffer without the reducing agent. Twenty five micrograms (i.e., 25 μg proteins) were loaded into a 4% stacking gel and run on a 12% acrylamide gel (PROTEAN^®^ Tetra Vertical Electrophoresis Cell) for 1 h at 120 V, as reported in previous work [11].

Prior to the mass spectrometry analysis, the proteins were heat denatured, reduced with dithiothreitol, alkylated with iodoacetamide, and digested with Sigma Trypsin (T-1426) at a ratio of 40 µg/mg protein. The trypsin hydrolysates were filtered through a 0.22 μm membrane followed by the injection of 0.4 µg into a 6550A iFunnel Q-TOF LC/MS (Agilent Technologies, Santa Clara, CA, USA). The conditions of analysis were as described in a recent work [11]. Peak lists from the MS/MS data were analyzed using the Mascot™ software (Matrix Science, London, UK, http://www.matrixscience.com). The sequences were matched to those available in the NCBInr database.

### 2.4. Protein Hydrolysis and Dialysis

Proteins from the control (i.e., no sonication), ultrasonic cleaner bath, or probe-type ultrasound-treated brans were hydrolyzed with either Flavourzyme, Papain, or Alcalase. In each case, 1.5 g of proteins was dissolved in 20 mL of water. Appropriate amounts of protease were added to ensure a 2% enzyme to substrate ratio (*w/w*). The pH and temperature conditions were as follows: Flavourzyme: pH 7.0 and 50 °C; Papain: pH 7.0 and 60 °C; Alcalase: pH 8.0 and 60 °C. All the digestions were performed for 3 h and terminated by heating in a hot water bath at 85 °C for 5 min. Once cooled to room temperature, they were centrifuged at 8000× *g* (15 min, 4 °C) to collect the hydrolysates (i.e., supernatants).

The dialysis was performed using a cellulose ester Spectra/Por^®^ (Fisher Scientific, Nepean, ON, Canada) membrane with a 100–500 Da molecular weight cut-off. The membranes were prepared by soaking in water to remove the storage buffer (0.05% sodium azide). The prepared membranes containing hydrolyzed protein solutions were immersed in water and gently stirred overnight (approximately 14 h). Solutions within the membranes were freeze dried, and their protein content was determined as previously reported [12].

### 2.5. Ultra-Violet and Fourier Transform Infrared (FT-IR) Aanalysis of Hydrolysates

Protein hydrolysates (1 mg/mL) were made in 75 mM of potassium phosphate buffer at pH 7.4. A volume of 200 µL (triplicates) was transferred to a clear microplate (Epoch^®^ BioTek^®^, Fisher Scientific, Nepean, Ontario, Canada). The spectra were recorded from 200–400 nm in addition to a single read at 280. The FT-IR spectra were obtained using the MB100 Arid-Zone™ Spectrometer (ABB Bomen Inc., Thunder Bay, Ontario, Canada), with a scanning rate of 19 scans/min and a resolution of 4 [11]. The data were analyzed using the GRAMS™ 32 Software (Thermo Fischer Scientific, Mississauga, Ontario, Canada). For the FT-IR analysis, the hydrolyzed proteins (1.0 mg) were mixed with KBr (400 mg) and transformed into pellets before recording the spectra from 600 to 4000 cm^−1^.

### 2.6. Analysis of Free Thiols and Amino Acids

The free thiol content of the hydrolyzed proteins was determined based on their reaction with 5,5’-dithio-*bis*-(2-nitrobenzoic acid) [13]. The hydrolysates (5 mg/mL), cysteine standards (0.0625–1.0 mM), and the Ellman’s reagent (5,5’-dithio-*bis*-(2-nitrobenzoic acid), 4 mg/mL) were prepared in buffer (0.1 M Tris, 1 mM EDTA, pH 8.0 with 8 M urea and 1% SDS). To each sample (300 µL), 20 µL of Ellman’s reagent was added and the sample was mixed and incubated at room temperature for 15 min. The absorbance was measured at 412 nm using an Epoch Microplate reader (BioTek, Vermont, USA), and the free sulfhydryl groups were determined using the standard curve and reported as µM SH/g protein. The free amino acid content was determined based on a previous study [14]. One hundred fifty microliters of each hydrolysate (0.2 mg/mL, 75 mM potassium phosphate buffer pH 7.4) or serine standards (0–140 μg/mL) were mixed with 150 μL of 0.5% ninhydrin in water and heated (100 °C, 30 min). They were diluted 1:5 with water before an absorbance reading at 570 nm.

### 2.7. Zeta Potential and Hydrophobicity Assays

The zeta potential was measured using a Zetasizer Nano Series Zen3600 (Malvern Instruments Ltd., Malvern, UK) with a Millex^®^ GP Filter and a 0.22 µM PES membrane (EMD Millipore Corporation, Bedford, MA, USA). The protein hydrolysates were analyzed at 2.5 mg/mL in water and their potential was recorded and reported in millivolts. The hydrophobicity was determined at seven concentrations 0.00125–0.03% in 0.01M phosphate buffer (pH 7) which are common in the literature [15]. For each concentration of the hydrolysate, 2 mL of was mixed with 10 μL of 8.0 mM 8-anilio-1-naphthalene sulfonate (ANS), followed by a fluorescence measurement at the excitation and emission wavelengths of 390 and 470 nm, respectively (Synergy H1 Microplate Reader, BioTek, Winooski, VT, USA). The fluorescence intensity was calculated using the following equation: , where FI is the fluorescence intensity, F_1_ is the fluorescence of hydrolysate and F_0_ is the fluorescence of the blank (phosphate plus ANS). The initial slope of the line from the fluorescence intensity (FI) versus protein concentration was used as the index of hydrophobicity, *H_0_*.

### 2.8. Antioxidant Assays

Three assays were used to assess the capacity of the protein hydrolysates to prevent the oxidative damage associated with three common reactive oxygen species (ROS). The peroxyl radical (ROO•) scavenging power was determined based on the oxygen radical absorbance capacity (ORAC) assay [16]. The hydrolysates were prepared at a concentration of 0.1 mg/mL The fluorescence measurements were performed as described in the above reference, and the ORAC values were reported as µM Trolox equivalents (TE)/g of hydrolysate. The hydroxyl radical (HO•) scavenging activity was measured by mixing in this order: hydrolysates (50 µL, 1.0 mg/mL) or phosphate buffer (50 µL, 75 mM, pH 7.4) with 1,10-phenanthroline (50 µL, 3 mM), FeSO_4_·7H_2_O (50 µL, 3 mM), and finally 0.03% aqueous H_2_O_2_ (50 µL). The mixtures were incubated at 37 °C for 1 h, after which the absorbance was measured at 536 nm (Epoch Microplate Reader, BioTek, Winooski, VT, USA) to measure the percent inhibition of the formation of HO• radicals [17]. The autoxidation reaction of pyrogallol was used for the determination of the superoxide anion radical (O_2_^•−^) scavenging power [12]. The hydrolysates were analyzed at 0.5 mg/mL, and the kinetic rates at 420 nm were used to calculate the O_2_^•−^ scavenging activities as percentages of the control rate. For each test, the natural tripeptide glutathione at the same concentration as the sample was used for comparison.

### 2.9. Alpha-Amylase and Dipeptidyl Peptidase-4 Inhibition Assays

The α-amylase inhibitory activity was determined based on a reported procedure [18]. Hydrolyzed proteins (1 mg/mL), acarbose (1 mg/mL, positive control), and α-amylase (1 U/mL) were prepared in 0.02 M sodium phosphate buffer containing 6 mM of NaCl, pH 6.8. Aliquots (100 µL) of hydrolysates or acarbose were pre-incubated with 100 µL of α-amylase at 37 °C for 5 min before the addition of 100 µL of substrate (1% starch in buffer) and a further 10 min incubation. The sample blanks (100 µL hydrolysate + 100 µL buffer) and negative controls (100 µL buffer + 100 µ α-amylase) were analyzed at the same time. To terminate the reaction, 200 µL of DNS reagent (1% 3,5-dinitrosalycilic acid, 12% sodium potassium phosphate tartrate in 0.04 M NaOH) was added, then heated in a boiling water bath for 5 min, after which it was placed on ice to cool. Once at room temperature, it were diluted (1:1) with buffer. The product formed between the glucose and DNS was measured at 540 nm and used to calculate the inhibitory effect of hydrolysates. The DPP-4 inhibition assay was performed using a kit (BML-AK499) from Enzo^®^ Life Sciences (Farmingdale, NY, USA). The procedure provided with the assay was followed. The stock concentrations of hydrolysates were 5 mg/mL (final assay concentration 1.25 mg/mL), while those of the DPP-4 enzyme, H-Gly-Pro-pNA substrate, and control were as specified in the kit manual. The absorbances at 405 nm were read for 60 min to calculate the DPP-4 inhibition as pmol/min.

### 2.10. Culture and Maintenance of NCI-H716 Cells

Human colorectal adenocarcinoma cells NCI-H716 (ATCC CCL^®^-251™, from Cedarlane® (Burlington, ON, Canada) were plated (10 cm) in Roswell Park Memorial Institute (RPMI) 1640 Medium (ATCC^®^ 30-2001) supplemented with 10% fetal bovine serum (ATCC^®^ 30-2021) both from Cedarlane® (Burlington, ON, Canada) and 2 mM of L-glutamine, as reported previously [19]. The incubation was at 37 °C in a Forma^TM^ Series II Water Jacketed CO_2_ (5%) Incubator (ThermoFischer Scientific, Franklin, MA, USA). The cells were fed every two days by replacing 50% of the old media with fresh ones.

### 2.11. Preparation of NCI-H716 Cells forBioassays

The cells were plated following the literature procedures [19,20]. Matrigel (Extracellular matrix (ECM), Sigma E1270) was thawed at 4 °C and diluted 10 times with cold (4 °C) Hank’s Balanced Salt Solution (HBSS; 0.4 g/L potassium chloride, 0.06 g/L potassium phosphate monobasic, 0.35 g/L sodium bicarbonate, 8.0 g/L sodium chloride, 0.048 g/L sodium phosphate dibasic, and 1.0 g/L D-glucose). Then, 50 µL was transferred to the wells of pre-cooled 96-well plates and allowed to gel at 37 °C for 1 h. Cells (5 × 10^4^ cells in 100 µL) were added to each well and left to grow for 48 h. The media were carefully removed and the cells were washed twice with 200 µL of HBSS. Protein hydrolysates (200 µL) prepared at 0.2 to 1.0 mg/mL (final concentrations) in Kreb’s Ringer Bicarbonate buffer (119 mM sodium chloride, 4.82 mM potassium chloride, 1.25 mM magnesium sulfate, 1.24 mM monosodium phosphate, 25 mM sodium bicarbonate, 2.0 mM HEPES, 1.0 mM calcium chloride), pH 7.4, and glucose (standard, prepared at 200 mM) were added and left to incubate at 37 °C for 2 h.

### 2.12. Effect of Hydrolysates on the Viability of Cells

The cell viability of NCI-H716 cells in the presence and absence of hydrolysates was determined based on the mitochondrial activity [21]. Specifically, after treatment with hydrolysates as described above, 5 µL of 3-(4,5-dimethylthiazol-2-yl)-2,5-diphenyltetrazolium bromide (MTT) solution (5 mg/mL in phosphate buffered saline (PBS)) was added to each well and further incubated for 1 h at 37 °C. At the end of the incubation, the reagent was removed from each well, followed by the addition of 100 µL of dimethyl sulfoxide. The viability, calculated as a percentage of the control, was determined by measuring the absorbance at 570 nm with a background subtraction at 630 nm (Cytation 5 Imaging Reader, BioTek, Winooski, VT, USA).

### 2.13. Glucagon-Like Peptide-1 (GLP-1) Secretion

In cells treated with hydrolysates for 2 h or in control cells, the supernatants were collected with the anti-protease phenylmethylsulfonyl fluoride (PMSF) solution (50 µg/mL final concentration) and frozen at −80 °C until further analysis. The GLP-1 secretion was determined using an ELISA kit (EGLP-35K, EMD Millipore Corporation, Bedford, Massachusetts, USA) according to the manufacturer’s instructions. Briefly, the samples were plated onto the antibody-coated plate for 24 h and a detection conjugate was added to bind the GPL-1 present in the samples, which was followed by the addition of the substrate. The fluorescence was measured at the excitation/emission wavelengths of 355 and 460 nm, respectively (Cytation 5 Imaging Reader, BioTek, Winooski, VT, USA), and used to calculate the concentration of GLP-1.

### 2.14. Statistics

All the experiments were performed in triplicate, except for mass spectrometry, SDS-PAGE, and FT-IR. The statistical analyses were performed using SAS^®^ Studio Online (© 2019 SAS Institute Inc., Cary, NC, USA). The results are expressed as mean ± the standard deviation. The results were analyzed by a one-way analysis of variance (ANOVA) and least significant difference (LSD) tests (*p* < 0.05). The function Regress was used to obtain linear correlations between data from different assays.

## 3. Results and Discussion

### 3.1. Characterization of Isolated Proteins

Three procedures—ultrasonic bath (UB), ultrasonic probe (UP), and the control (CTL, no sonication)—were used to pre-treat medium oat brans before extraction. The soluble protein contents of the extracted proteins were 90.1%, 89.4%, and 93.5% for the CTL, UB, and UP samples, respectively. The high protein contents indicate a high efficiency of the extraction procedures. The value of the CTL is similar to that of a previous study [11]. The isolated proteins were characterized using gel electrophoresis and mass spectrometry. The SDS-PAGE data (Suppl. Figure 1) showed that, in general, the three protein samples had similar molecular weight distributions, with the only difference being in the intensity of the polypeptide bands. In the absence of the reducing agent, the intensity of the major band at 50–60 kDa (11S and 12S globulins) was less intense in the brans treated with ultrasounds. In the reduced samples, the intensity of the two major bands around 35 and 20 kDa was less intense in the control sample. This is likely because the ultrasonic treatment caused conformational changes that facilitated the access of disulfide bonds to the reducing agent. Greater changes were associated with the use of ultrasonic probes. The disulfide bonds in globulins are cleaved by the reducing agent (2-mercaptoethanol) to produce acidic (35–40 kDa) and basic (20–25 kDa) subunits [10]. The effect of sonication on the molecular weight distribution of proteins by SDS-PAGE depends on the food source. Comparable to this work, for example, no change was found in walnut proteins, while changes in the molecular weight distribution of albumin and jackfruit seed proteins was found after sonication [9].

In addition to gel electrophoresis, the polypeptide composition of the isolate proteins was determined using mass spectrometry. Peaks listed from the MS/MS data of the trypsin digests were analyzed using the Mascot^TM^ software. The determined sequences were matched with those of oat (i.e., *Avena sativa*) proteins present in the NCBInr database. The software uses sequences of peptides to calculate the percentage coverage. In total, 14 polypeptides (Table S1) were identified. Of the polypeptides present in all the three isolates, four were 12S globulins-type, two were 11S globulins-type, and five were avenins-type proteins. Two polypeptides, vromindoline and avenin-E, were detected in the isolated proteins from two sonicated bran samples, while tryptophanin was only obtained found when the ultrasonic probe was used to treat the bran. The difference is likely because the cavitation is unevenly distributed in the ultrasonic bath and also because probe-type ultrasonic systems have a much higher intensity [22]. Vromindolines are starch-bound proteins that can contribute to an up to 50 % reduction in the oat grain hardness [23]. Tryptophanin proteins also contribute to the oat grain softness, because they are bound to lipids [24]. It can be concluded that the sonication treatments disrupted the flour matrix to release these proteins. There are many genes that encode for globulin and avenin proteins, and this results in slight differences in amino acid sequences and molecular weights [10]. The higher percentage coverage of the 11S and 12S globulins (53–59 kDa) compared to the avenins is indicative of their higher abundance, and this correlated with the SDS-PAGE data.

### 3.2. Hydrolysis of Proteins and Characterization of Hydrolysates

Each protein isolate was hydrolyzed with three food-grade proteases (Flavourzyme, Papain, Alcalase) to evaluate the effect of both the extraction procedure and the specificity of the protease on the physicochemical and biological functions. Independently of the extraction procedure or the nature of the proteases, Flavourzyme hydrolysates had the lowest protein contents of (23.8–40.5%), while there was a similarity in the protein contents of Papain (56.0–61.7%) and Alcalase (49.9–54.8%) hydrolysates (Figure S2a). The lower soluble protein content of the Flavourzyme hydrolysates is due to its higher proteolytic activity, which translated into higher free amino contents: 27.4–32.8%, compared to 4.6–7.5% for hydrolysates produced with Papain and Alcalase (Figure S2b). Other works have reported greater free amino acid contents due the action of Flavourzyme compared to other proteases [25,26]. This is because Flavourzyme contains endo- and exo-peptidase activities, while Papain and Alcalase only contain endopeptidases [12]. The exopeptidase activity is associated with the release of free amino acid from the C- or N-terminal of proteins.

The ultrasonic pre-treatments of brans resulted in greater amounts of sulfhydryl or thiol groups (SH) in the Flavourzyme hydrolysates, with the ultrasonic bath having the greatest effect (Figure S2c). The ultrasonic treatment has been found to disrupt tertiary structure of proteins, disrupt protein aggregates, or change the conformation of proteins [9,27]. In some cases, they can decrease sulfhydryl groups, as high intensity waves from the probe can, for example, generate ROS, which then oxidize SH groups [28,29]. In contract to the literature work, the changes in free thiols from this work are due to the action of the proteases and not to the ultrasounds. There was a reduction in SH in the Papain and Alcalase hydrolysates when the ultrasonic probe was used, but no change was associated with the ultrasonic bath treatment. It can be concluded that there was a difference in the tertiary structure of the isolated proteins that affected the accessibility of the peptide bonds to the same protease.

### 3.3. Zeta Potential and Hydrophobicity of Hydrolyzed Proteins

**Figure 1:**
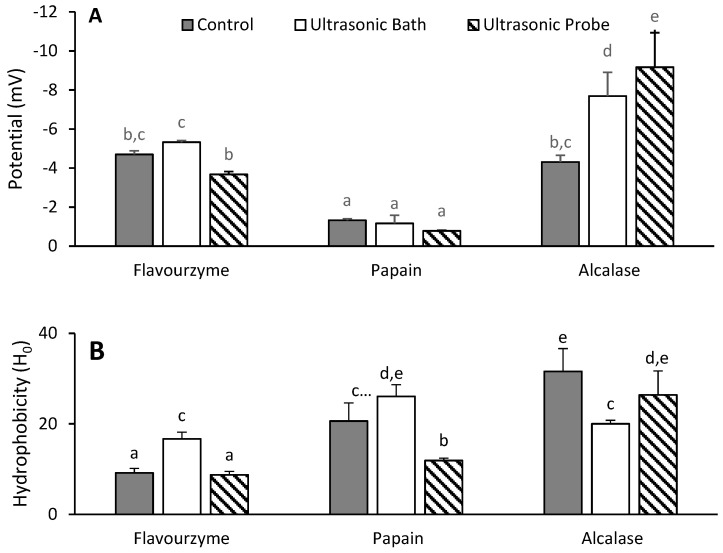
(**A**) Zeta potential (mV) of hydrolysates and (**B**) hydrophobicity index (H_0_) for protein hydrolysates, expressed as the initial slope of the relative fluorescence versus the concentration of protein. The letters above each bar represent significant differences as determined with a least significant difference (LSD) test in a one-way ANOVA (*p*-value < 0.05).

The hydrolysates were further characterized by measuring their surface hydrophobicity and charges (i.e., zeta potential)—two of the properties that can affect the biological function of peptides or proteins. All the hydrolysates had a negative zeta potential (Figure 1a), which means that they contained more negative amino acids than positive ones. The alcalase hydrolysates from sonicated oat brans had greater zeta potentials, −7.69 ± 1.21 mV for UB and −9.17 ± 1.76 mV for UP, while all the Papain hydrolysates had the smallest potentials (−0.78 to −1.32 mV). One would expect the zeta potential to change upon ultrasonic treatment because of the disruption of the protein structure, but whether this increases or decreases the surface charge upon hydrolysis seems, based on our data, to depend on the nature of the protease. This is illustrated by an increase in potential in the UP hydrolysates when Alcalase was used for hydrolysis. In contrast, a decreased potential was recorded when in the presence of Flavourzyme, while the treatment of the UP proteins with Papain had no effect on the potential.

Charged amino acids are polar, and their presence on the surface increases solubility and can also enhance the function of proteins or polypeptides [30]. An increase in zeta potential is then expected to be associated with a decrease in the surface hydrophobicity. This is partially confirmed by the hydrophobicity data of the protein hydrolysates (Figure 1b), as two of the Papain hydrolysates (CTL, UB) with the lowest zeta potential had a higher surface hydrophobicity than all the Flavourzyme hydrolysates and the UB sample hydrolyzed with Alcalase. The surface hydrophobicity of the control proteins was the most affected by the nature of the protease. Specifically, the hydrophobicity index (H_0_) was 9.2 ± 1.1, 20.6 ± 4.0, and 31.6 ± 5.1 after Flavourzyme, Papain, and Alcalase hydrolysis, respectively. In the case of UP sonication, H_0_ was only different for the Alcalase hydrolysate. Like the zeta potential, the surface hydrophobicity can provide insight into the structural changes of proteins or their hydrolysates in solution. There was no consistent pattern in the hydrophobicity of the tested hydrolysates; however, the low H_0_ of Flavourzyme hydrolysates for two of the proteases can be explained by their higher contents of free amino acids and lower protein contents (Figure S2a,b). Since Flavourzyme contains endo- and exo-proteases, more protein was hydrolyzed, meaning that there were fewer hydrophobic regions remaining. The relatively high H_0_ of Papain and Alcalase hydrolysates relative to those of Flavourzyme is because both Papain and Alcalase share some preference for cleavage at bonds before or after hydrophobic residues [12]. In contrast, the exo-peptidase activity has been used for the removal of hydrophobic amino acids at the N-terminus of proteins, thereby decreasing their bitterness [31].

### 3.4. Spectroscopic Characterization of Hydrolyzed Proteins

**Figure 2:**
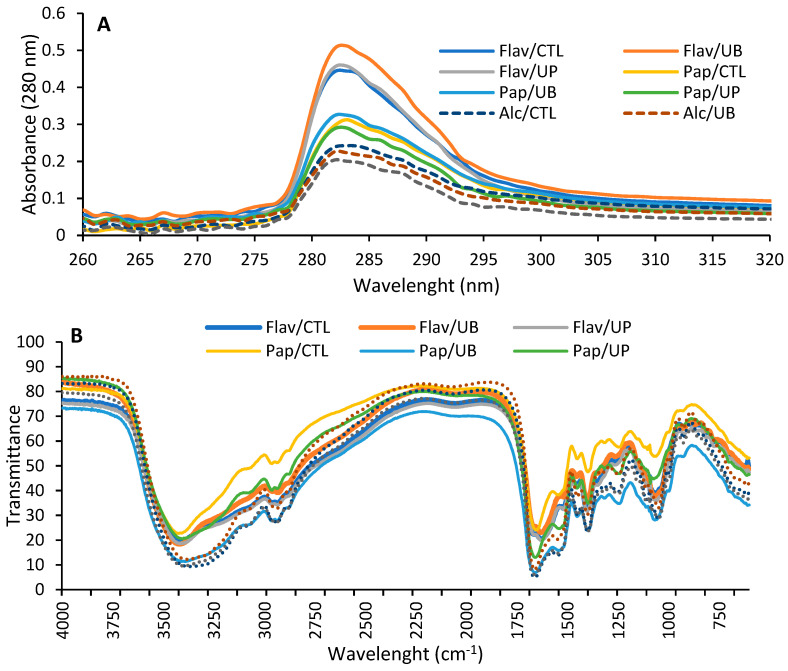
Spectroscopic spectra of hydrolysates from proteins extracted in control (CTL), ultrasonic bath (UB), or ultrasonic probe (UP) conditions. (**A**) UV spectra from 260 to 320 nm; (**B**) FT-IR recorded between 600 and 4000 cm^−1^. Proteins were hydrolyzed with Flavourzyme (Flav), papain (Pap), or alcalase (Alc).

The UV absorbance spectra and Fourier-Transform infrared spectra (Figure 2) were measured to characterize the functional groups present in the hydrolysates. In Figure 2a, the UV spectra shows the relative intensity of chromophores from phenylalanine, tyrosine, and tryptophan that had an absorption of between 260 and 322 nm. This is useful for assessing changes in the environment of aromatic amino acids [32]. An analysis of the UV spectra showed no contribution from of phenylalanine, which absorbs at 260–265 nm [33]. The UV spectra can be grouped into three based on the proteases, as there is little effect of the extraction procedure. Flavourzyme hydrolysates had the highest intensities, likely because of the pronounced structural modifications due to its endo- and exo-peptidase activities causing greater the exposure of aromatic residues. This is supported by their higher degree of hydrolysis (i.e., higher free amino acids). In a recent work as well, the UV spectra were used to also demonstrate the increased exposure of aromatic amino acids upon the treatment of egg proteins with proteases [34].

In Figure 2b, the FT-IR spectra of hydrolysates are similar and displayed the characteristic proteins bands. All the spectra had bands around 1630–1660 cm^−1^ and 1540–1550 cm^−1^, which are characteristic of amide I (C=O bending vibrations) and amide II (N-H deformation and C-N stretching vibrations), respectively [11,35]. The amide A and amide B bands observed at 3380–3400 cm^−1^ and 3030–2840 cm^−1^ are representative of the N-H and alkyl group stretching vibrations but might contain contributions from the hydroxyl groups. The hydrolysates also had peaks at ~1060 cm^−1^, corresponding to alcohol groups, which might come from residual polysaccharides remaining in the sample [36]. The absorption bands in these spectra are similar to previous findings on proteins from oat milling fractions [11].

### 3.5. Antioxidant Activities of Hydrolyzed Proteins

**Figure 3:**
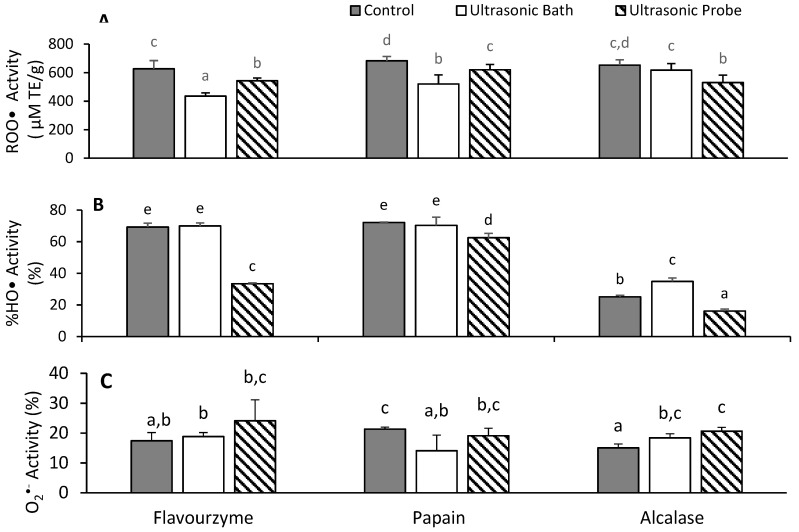
(**A**) Peroxyl radical (**A**), Hydroxyl radical (**B**), and Superoxide radical (**C**) scavenging activities of hydrolysates. Data are expressed as the mean of triplicates ± the standard deviation. The letters above each bar represent significant differences as determined with a least significant difference (LSD) test in a one-way ANOVA (*p*-value < 0.05).

One of the functions of hydrolyzed food proteins is the role as antioxidants. As such, this work assessed the effect of sonication on the peroxyl (ROO•), hydroxyl (HO•), and superoxide (O_2_^•−^) radical scavenging properties of oat protein hydrolysates (Figure 3). Sonication caused a statistically significant reduction in the peroxyl radical scavenging activity for all the protein hydrolysates (Figure 3a). The control hydrolysates scavenged ROO•, with oxygen radical absorbance capacity (ORAC) values of 627.17 ± 57.36, 682.90 ± 30.21, and 652.67 ± 36.94 µM TE/g when the protease was Flavourzyme, Papain, and Alcalase, respectively. The Flavourzyme and Papain hydrolysates from brans pre-treated with ultrasonic bath (UB) had lower activities than those from corresponding brans pre-treated with the ultrasonic probe (UP), while for Alcalase the hydrolysate from UP-treated brans had lower activities relative to the UB-treated brans. In the HO• test, the most effective hydrolysates with quenching values of 62.5–72.1% were generated using Flavourzyme and Papain (Figure 3b). The effect of protease was very minimal on HO•, except for UP-Flavourzyme hydrolysate, which showed about half the activity of the two other proteases. The Alcalase hydrolysates were much less effective in their capacity to scavenge HO• radicals, with quenching values of 16.1–34.9%. The O_2_^•−^ radical scavenging activities ranged from 14.1 to 24.1% (Figure 3c). When the protease was Alcalase, the UB pre-treatment increased the O_2_^•−^ activity relative to the CTL, while the UP pre-treatment increased the activity beyond that of UB. A similar trend was found in the case of Flavourzyme, but not Papain.

Overall, the ultrasonic pre-treatment of oat brans decreased or had no effect on the radical scavenging activity of the oat bran protein hydrolysates. There was no linear correlation with the physicochemical properties. Alcalase hydrolysates with higher hydrophobicity index and low concentration of SH groups had the least HO• scavenging activities. This is likely because SH is a good proton-donating functional group and lower concentration in a sample can result in a reduction of HO• scavenging activities, meanwhile, the HO• activity is also dependent on the ability of peptide fragments to chelate ferrous ions [17]. A recent study reported an increase in the antioxidant activities of various soy protein hydrolysates, which was partly explained by the content of SH functional groups [37]. Flavourzyme hydrolysates had the highest content of aromatic amino acids (Figure 2a), however its antioxidant activity did not differ, likely because the sequence of peptides present in the hydrolysates was more important. Other studies have shown a greater importance of peptide sequences over their aromaticity [4,7].

### 3.6. Inhibition of Alpha-Amylase and Dipeptidyl Peptidase-4

**Figure 4:**
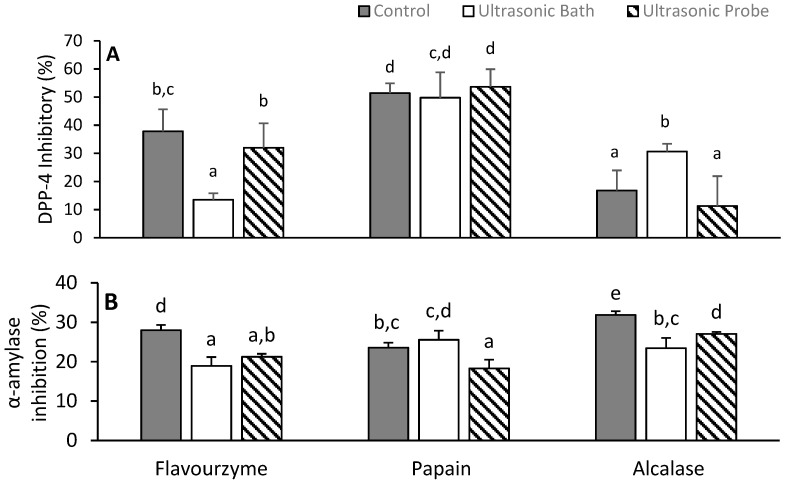
(**A**) Dipeptidyl peptidase-4 (DPP-4) and (**B**) α-Amylase inhibition by protein hydrolysates at 1 mg/mL and 1.25 mg/mL, respectively. Data are expressed as the mean of triplicates ± the standard deviation. The letters above each bar represent significant differences as determined with a least significant difference (LSD) in a one-way ANOVA (*p*-value < 0.05).

Protein hydrolysates showed α-amylase and DPP-4 inhibitory activities (Figure 4). These enzymes play important roles in glucose digestion and insulin secretion, and thus their inhibition is a measure of antidiabetic properties. Depending on the protease, the ultrasonic pre-treatment of brans either maintained or reduced the inhibitory activity. In one case, however, the UB pre-treatment increased the DPP-4 inhibitory activity of the Alcalase hydrolysate to 30.6% ± 4.7% compared to 16.8 ± 3.4% for the control (Figure 4a). Papain hydrolysates, regardless of the extraction procedure, showed the highest DPP-4 inhibitions (47.7–53.6%) relative to the other hydrolysates. The action of different proteases on the same protein isolate produced different DPP-4 inhibitions. Meanwhile, each type of sonication produced comparable results in most cases. The α-amylase inhibitory properties of protein hydrolysates were affected by the method used to extract the proteins. Except for the UB Papain hydrolysate, the hydrolysates of proteins from ultrasonic pre-treated brans displayed a reduced α-amylase inhibition relative to the control (Figure 4b). The α-amylase inhibition data (18–32%) are similar to some literature values [38].

The intensity and duration of sonication has been reported to influence the ability of protein hydrolysates to inhibit enzymes, such as angiotensin-converting enzyme [39], but no data could be found concerning the effect of such processes on the inhibition of DPP-4 activities. There is, however, literature data on the inhibition of DPP-4 by hydrolysates from proteins obtained without ultrasonic treatments. Some of the literature data, like those of quinoa hydrolyzed proteins (IC_50_ of 0.98 mg/mL) [40] and amaranth glutelins hydrolysates (IC_50_ 1.2–2.0 mg/mL) [7], have DPP-4 inhibitions that are comparable to values obtained in this work for papain hydrolysates. Higher inhibitions were reported for oat globulin and glutenin fractions after simulated gastrointestinal digestion [41]. Data also exists on the inhibition of α-amylase by protein hydrolysates cereals such rice brans [3], and the value varies depending on the hydrolysis conditions. The mechanism of inhibition has not been elucidated, but kinetic studies as summarized in a review [8] have shown that the inhibition of α-amylase by food peptides often occurs through competitive binding between polysaccharides and peptides, primarily via their aromatic amino acids.

### 3.7. Effect of Hydrolyzed Proteins on Cellular Viability and the Secretion of Glucagon-Like Peptide in NCI-H716 Cells

**Figure 5:**
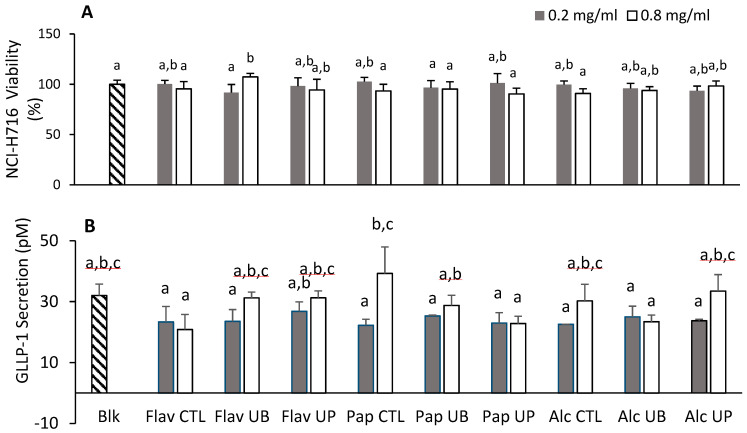
(**A**) Cell viability of NCI-H716 cells as determined by an 3-(4,5-dimethylthiazol-2-yl)-2,5-diphenyltetrazolium bromide (MTT) assay; (**B**) GLP-1 secretion stimulated by hydrolysates. Cells were plated at 5 × 10^4^ cells in a 96-well microplate with Matrigel for 24 h and were treated with hydrolysate for 2 h. Data are expressed as the mean of triplicates ± the standard deviation. The letters above each bar represent significant differences, as determined with an LSD test in a one-way ANOVA (*p*-value < 0.05).

The NCI-H716 cell line is currently the only human model available for the in vitro study of GLP-1; however, there are two other GLP-1-producing cell lines from mice—namely, GLUTag and STC-1 [19]. The cytotoxicity of hydrolysates was determined based on the reduction of the MTT tetrazolium salt to formazan by mitochondrial oxidoreductase enzymes (MTT assay). The concentration of formazan is proportional to the number of viable cells. The hydrolysates were tested at concentrations of 0.2 to 1.0 mg/mL. Data for the lowest and one higher (0.8 mg/mL) concentration are presented in Figure 5a. There was no significant decrease in the viability of cells treated with the hydrolysates relative to the untreated cells.

The secretion of GLP-1 was measured in response to treatment with hydrolysates at 0.4 mg/mL and 0.8 mg/mL (Figure 5b). The range of GLP-1 secreted for all samples was 20.85–39.25 pM. The positive control (cells treated with glucose) had a GLP-1 concentration of 42.89 pM compared to the negative control (i.e., media only). Except for Papain UP and Alcalase UB hydrolysates, an overall dose-dependent response was observed; however, it is not large enough to be statistically significant or increase the GPL-1 secretion beyond the value of the control cells. Figure 5b shows that testing concentrations greater than 0.8 mg/mL may have resulted in a significant increase in the GPL-1 secretion. This is likely because the casein proteins at concentrations of 5 mg/mL significantly increased the GPL-1 secretion, while whey proteins at the same concentrations had no effect [42]. Future tests could focus on higher concentrations of hydrolyzed oat proteins and sub-fractions. The exact mechanism by which food peptides control the GLP-1 secretion is unknown. Food peptides have been shown to affect gene expression and the activation of bile acid and calcium receptors [20]. The determination of the peptidomic profiles of the hydrolysates is necessary to determine their possible contribution to the activity.

## 4. Conclusions

The sonication of oat brans resulted in the presence of polysaccharide and lipid-bound polypeptides in the isolated proteins. Meanwhile, when the proteins were enzymatically hydrolyzed, their physicochemical properties were more affected by the nature of the protease than the method used to extract the proteins. Papain hydrolysates had the highest DPP-4 inhibition activities, which might be related to their low surface potential. Both Papain and Alcalase hydrolysates have potential to increase the cellular secretion of GPL-1 in NCI-H716 cells; however, testing at higher concentrations and the determination of peptide profiles is necessary to determine the contribution of individual molecules. Additionally, knowledge of the stability of the peptides in the hydrolysates is important to better assess and understand their role in biological systems.

## Supplementary Materials

The following are available online at https://www.mdpi.com/2076-3921/9/6/557/s1, Figure S1: SDS-PAGE gel electrophoresis of isolated proteins in the absence and in the presence of a reducing agent. Samples (25 μg) were run on a 12% resolving gel and 4% stacking gel for 1 hour at 120V and. Samples control (CTL) and brans pre-treated with ultrasonic bath (UB) or ultrasonic probe (UP) ultrasounds, Figure S2: (A) Soluble protein contents of hydrolysates in weight percentages; (B) Concentration of free amino acids (FAA, µg/g hydrolysate); (C) Concentration of free sulfhydryl or thiol (SH) groups (µg/g protein). Data are means of triplicates ± standard deviation. Value with different letters are significantly different as determined by a one-way ANOVA coupled with a least significant difference (LSD) test (*p*-value < 0.05), Table S1: Composition of extracted proteins from oat brans. Each protein isolate was analyzed using LC-MS/MS after digestion by trypsin. Myoglobin was used as a control, and the sequences were analyzed using Mascot^TM^ Software. (-) indicates an absence of protein. Proteins are from control (CTL) and brans pre-treated with ultrasonic bath (UB) or ultrasonic probe (UP) ultrasounds.
