# Supplementary material for: Antioxidant, Physicochemical, and Cellular Secretion of Glucagon-Like Peptide-1 Properties of Oat Bran Protein Hydrolysates
Source: Antioxidants (Basel). 2020 Jun 26;9(6):557. doi: 10.3390/antiox9060557 (PMC7346174; doi:10.3390/antiox9060557)
Supplement: Supplementary file 1 [file antioxidants-09-00557-s001.zip › Walters et al. Suppl. Table.pdf]

Suppl. Table 1: Composition of extracted proteins from oat brans. Each protein isolate was analyzed using LC-MS/MS after digestion by trypsin. Myoglobin was used as a control, and the sequences were analyzed using Mascot^{TM} Software. (-) indicates an absence of protein. Proteins are from control (CTL) and brans pre-treated with ultrasonic bath (UB) or ultrasonic probe (UP) ultrasounds.

NCBInr protein i.d.	Protein name	MW (Da)	Sample	Coverage (%)
P12615.1	12S Seed Storage Globulin 1	58964	CTL	33
			UB	34
			UP	31
P14812.1	12S Seed Storage Globulin 2	59038	CTL	34
			UB	36
			UP	35
CAA52764.1	11S Globulin	59768	CTL	33
			UB	30
			UP	30
CAA54152	12S Globulin	53737	CTL	30
			UB	29
			UP	30
CAA54153.1	12S Globulin	58531	CTL	25
			UB	25
			UP	25
CAA52763.1	11S Globulin	62223	CTL	31
			UB	27
			UP	25
P27919.1	Avenin	24670	CTL	25
			UB	25
			UP	38
CBL51494.1	Avenin protein	26116	CTL	24
			UB	24
			UP	24
CBL51489.1	Avenin protein	26391	CTL	26
			UB	26
			UP	26
AAA32716.1	Avenin	25911	CTL	8
			UB	8
			UP	-
AFJ04424.1	Vromindoline	17007	CTL	-
			UB	19
			UP	29
AAB32025.1	Alcohol soluble avenin-3	23718	CTL	-
			UB	14
			UP	34
Q09114.1	Avenin-E	21479	CTL	-
			UB	19
			UP	35
ABU39831.1	Tryptophanin	16461	CTL	-
			UB	-
			UP	33
