# Supplementary material for: Antioxidant, Physicochemical, and Cellular Secretion of Glucagon-Like Peptide-1 Properties of Oat Bran Protein Hydrolysates
Source: Antioxidants (Basel). 2020 Jun 26;9(6):557. doi: 10.3390/antiox9060557 (PMC7346174; doi:10.3390/antiox9060557)
Supplement: Supplementary file 1 [file antioxidants-09-00557-s001.zip › Walters et al. Suppl_Figures.pdf]

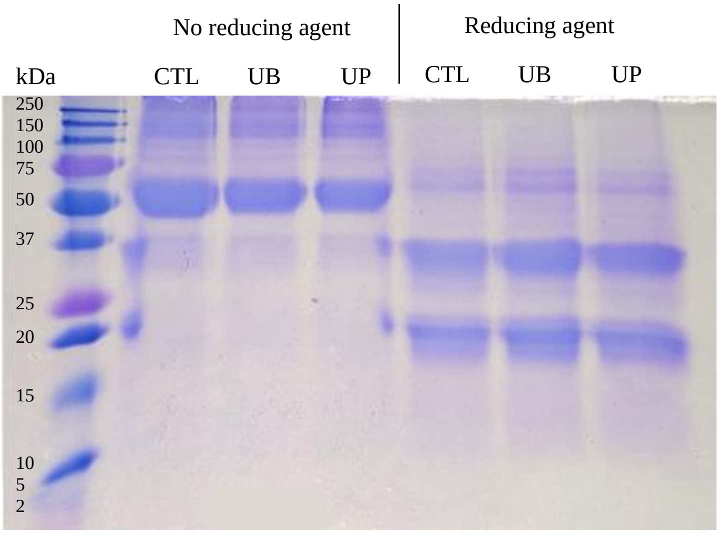

Suppl. Figure 1: SDS-PAGE gel electrophoresis of isolated proteins in the absence and in the presence of a reducing agent. Samples (25 μg) were run on a 12% resolving gel and 4% stacking gel for 1 hour at 120V and. Samples control (CTL) and brans pre-treated with ultrasonic bath (UB) or ultrasonic probe (UP) ultrasounds.

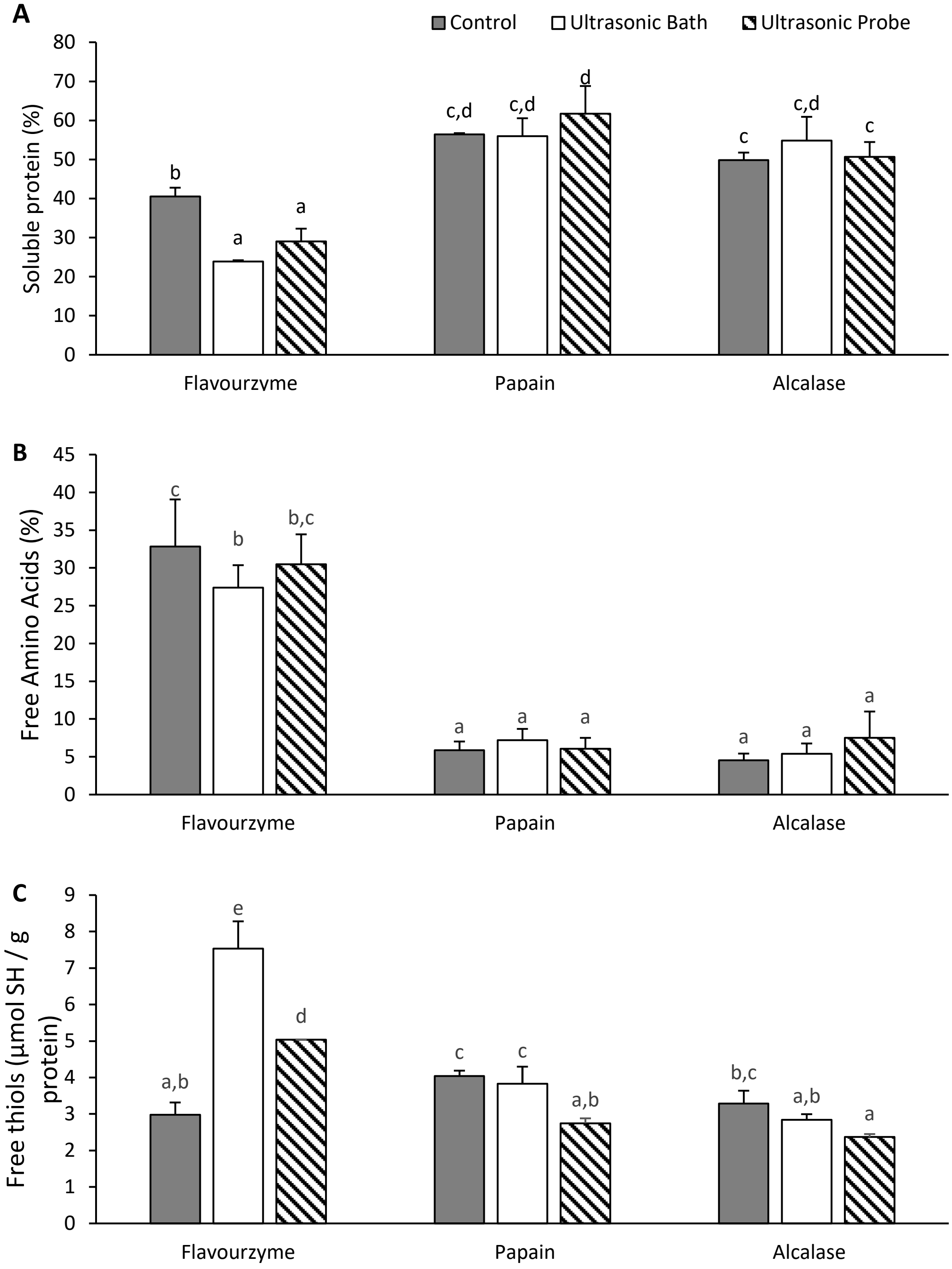

Suppl. Figure 2: A) Soluble protein contents of hydrolysates in weight percentages; B) Concentration of free amino acids (FAA, µg/g hydrolysate); C) Concentration of free sulfhydryl or thiol (SH) groups (µg/g protein). Data are means of triplicates ± standard deviation. Value with different letters are significantly different as determined by a one-way ANOVA coupled with a least significant difference (LSD) test (p-value < 0.05).
